# Transferrin-Conjugated SNALPs Encapsulating 2′-O-Methylated miR-34a for the Treatment of Multiple Myeloma

**DOI:** 10.1155/2014/217365

**Published:** 2014-02-13

**Authors:** Immacolata Scognamiglio, Maria Teresa Di Martino, Virginia Campani, Antonella Virgilio, Aldo Galeone, Annamaria Gullà, Maria Eugenia Gallo Cantafio, Gabriella Misso, Pierosandro Tagliaferri, Pierfrancesco Tassone, Michele Caraglia, Giuseppe De Rosa

**Affiliations:** ^1^Department of Pharmacy, Federico II University of Naples, Via Domenico Montesano 49, 80131 Naples, Italy; ^2^Department of Medical Oncology, Department of Experimental and Clinical Medicine, Magna Graecia University and T. Campanella Cancer Center, Salvatore Venuta Campus, 88100 Catanzaro, Italy; ^3^Department of Biochemistry, Biophysics and General Pathology, Second University of Naples, 80138 Naples, Italy

## Abstract

Stable nucleic acid lipid vesicles (SNALPs) encapsulating miR-34a to treat multiple myeloma (MM) were developed. Wild type or completely 2′-O-methylated (OMet) MiR-34a was used in this study. Moreover, SNALPs were conjugated with transferrin (Tf) in order to target MM cells overexpressing transferrin receptors (TfRs). The type of miR-34a chemical backbone did not significantly affect the characteristics of SNALPs in terms of mean size, polydispersity index, and zeta potential, while the encapsulation of an OMet miR-34a resulted in a significant increase of miRNA encapsulation into the SNALPs. On the other hand, the chemical conjugation of SNALPs with Tf resulted in a significant decrease of the zeta potential, while size characteristics and miR-34a encapsulation into SNALPs were not significantly affected. In an experimental model of MM, all the animals treated with SNALPs encapsulating miR-34a showed a significant inhibition of the tumor growth. However, the use of SNALPs conjugated with Tf and encapsulating OMet miR-34a resulted in the highest increase of mice survival. These results may represent the proof of concept for the use of SNALPs encapsulating miR-34a for the treatment of MM.

## 1. Introduction

MicroRNAs (miRNAs) are noncoding nucleic acids able to regulate basic biological functions and pathways essential to tumor development and progression [1]. In the last years, the development of miRNA-based anticancer therapies received growing attention. Some miRNAs, such as miR-34a, have a tumor suppressor activity and their reintroduction into diseased tissues can drive a therapeutic response [2]. The development of therapies based on miRNAs, as well as in general on nucleic acids, is hampered by several drawbacks, including cost and production of clinical grade material [3], degradation and inactivation by nucleases in plasma and cells [4], poor intracellular delivery [5, 6], rapid plasma elimination [7–9], and renal and dose-limiting hemodynamic toxicities [10]. For antisense oligonucleotides (ONs), which are furthest along the clinical development, nuclease sensitivity has been minimized through chemical modifications to the nucleic acid backbones and/or sugars [11], while hemodynamic toxicities have been reduced through the use of repeated, slow infusions (2 h) or continual infusion protocols up to 21 days [12]. The use of nanovectors for the delivery of nucleic acids represents a valid and widely investigated approach to overcome the previously described biopharmaceutical issues [11, 13]. This will be particularly critical for RNA agents on which modifications would be increasingly more complicated and costly. Conventional and cationic liposomes have been used extensively to increase the therapeutic index of a variety of ONs by changing their pharmacokinetic and pharmacodynamic characteristics [14–16]. However, the low ONs encapsulation efficiency (especially for neutral liposomes), the instability in serum, and the toxicity issues associated with the use of cationic lipid [13] limited the *in vivo* use of cationic liposomes. The use of an ionizable aminolipid into the lipid vesicles allowed facilitating the encapsulation of nucleic acids into the vesicle that can subsequently be brought at physiological pH with consequent increased stability in serum [17]. These nanocarriers, the so-called stable nucleic acid lipid vesicles (SNALPs), have shown promising results to deliver plasmids, antisense oligonucleotides, and small interfering RNAs (siRNA) *in vivo* [18–20].

Previously, our group developed SNALPs encapsulating miR-34a for the treatment of medulloblastoma cells [21]. The miR-34 is a family of noncoding RNAs directly regulated by the transcription factor p53 [22–24]. miR-34a activates the p53 oncosuppressor activity, by inhibiting cell growth, inducing apoptosis, and causing a senescence-like phenotype [25]. Several studies have confirmed that the miR-34 family is required for normal cell responses to DNA damage following irradiation *in vivo*.

Multiple myeloma is a hematologic malignancy, incurable in most cases, which needs development of novel therapeutic strategies [26]. Deregulated expression of miR-34a in MM patients had been evidenced [27], thus suggesting an interest for these molecules as antitumor therapeutic agents. In this light, a recent report by our group has demonstrated that miR-34a replacement strategies are active in controlling the proliferation of several MM cell lines in different animal models [28]. However, the optimization of the delivery of miR-34a is required in order to translate these models in the clinical setting.

The aim of this study was to develop SNALPs encapsulating miR-34a with enhanced delivery properties for MM. In particular, we investigated the possibility to encapsulate synthetic miRNAs, namely, wild type miR-34a and a completely 2′-O-methylated (OMet) miR-34a, into the SNALPs. Moreover, SNALPs encapsulating miR-34a were conjugated with transferrin (Tf), in order to achieve improved transfection efficiency and targeting types of tumor cells. Antitumor properties of the different miR-34a, encapsulated in SNALPs or Tf-conjugated SNALPs (Tf-SNALPs), were investigated in preclinical experimental model of MM.

## 2. Materials and Methods

1,2-Dioleyl-3-dimethylammonium propane (DODAP) and N-palmitoyl-sphingosine-1-{succinyl[methoxy(polyethylene glycol)2000]} (PEG_2000_-Cer_16_) were purchased from Avanti Polar Lipids. Distearoylphosphatidylcholine (DSPC) was kindly offered from Lipoid GmbH (Cam, Switzerland). Cholesterol (CHOL), human transferrin (Tf), sodium chloride, sodium phosphate, HEPES, citric acid, and sodium citrate were purchased from Sigma Aldrich (USA). Ethanol and other solvents were obtained from Carlo Erba Reagenti (Italy). MiR-34a wild type sequence obtained by miR.org database (5′-UGG CAG UGU CUU AGC UGG UUG U-3′) was directly synthesized by MWG (Germany). As control an oligonucleotide with scrambled sequence (miR-NC) was used (Life Technologies).

### 2.1. 2′-OMet Oligonucleotide Synthesis and Purification

The 2′-OMet Oligonucleotide were synthesized on a Millipore Cyclone Plus DNA synthesizer at 1 *μ*mol scale, using commercially available 5′-O-(4,4′-dimethoxytrityl)-2′-O-methyl-3′-O-(2-cyanoethyl-N,N-diisopropyl) RNA phosphoramidite monomers and 2′-OMet RNA SynBase CPG 1000/110 as solid phase support (Link technologies). The oligomers were detached from the support and deprotected by treatment with concentrated aqueous ammonia at 55°C for 12 h. The combined filtrates and washings were concentrated under reduced pressure, redissolved in H_2_O, analysed, and purified by anion-exchange high-performance liquid chromatography (HPLC) on a Nucleogel SAX column (Macherey-Nagel, 1000-8/46), using buffer A: 20 mM KH_2_PO_4_/K_2_HPO_4_ aqueous solution (pH 7.0), containing 20% (v/v) CH_3_CN, and buffer B: 1 M KCl, 20 mM KH_2_PO_4_/K_2_HPO_4_ aqueous solution (pH 7.0), containing 20% (v/v) CH_3_CN; a linear gradient from 0 to 100% B for 45 min and flow rate 1 mL/min were used. The purified oligomers were successively desalted by Sep-Pak C-18 cartridges (Waters) and characterized by ESI mass spectrometry.

### 2.2. Preparation of miR-34a Encapsulating SNALPs Conjugated or Not with Tf

SNALPs formulations were prepared by modified ethanol injection method. Briefly, lipid stock solutions were prepared in ethanol. Aliquots (0.4 mL total volume) of stock lipids (CHOL/DSPC/DODAP/PEG_2000_-Cer_16_ or CHOL/DSPC/DODAP/DSPE-PEG_2000_-Mal/PEG_2000_-Cer_16_) were added to a glass tube. In a separated tube, 0.2 mg of miRNA was dissolved in 0.6 mL of 20 mM citric acid at pH 4.0. The two solutions were warmed for 2-3 min to 65°C and the lipid solution was quickly added to the miRNA solution under stirring. The mixture was passed 5 times through 200 nm and 20 times through 100 nm polycarbonate filters using a thermobarrel extruder (Northern Lipids Inc., Vancouver, BC, Canada) maintained at approximately 65°C. Therefore, the preparation was dialyzed (3,5 kDa cutoff) against 20 mM citrate buffer at pH 4.0 for approximately 1 h to remove excess of ethanol, followed by further dialysis against HBS (20 mM HEPES, 145 mM NaCl, and pH 7.4) for 12–18 h to remove the citrate buffer and to neutralize the DODAP. Unencapsulated miRNA was removed by DEAE-Sepharose CL-6B column chromatography. A typical 1 mL formulation consisted of DSPC/CHOL/DODAP/PEG_2000_-Cer_16_ or DSPC/CHOL/DODAP/DSPE-PEG_2000_-Mal/PEG_2000_-Cer_16_ (25/45/20/10 or 25/45/20/5/5 mol/mol/mol/mol, resp.) and 0.2 mg of miR in a final solution. Tf was coupled on the preformed SNALPs vesicles containing DSPE-PEG-Mal in the lipid mix (SNALPs-Mal). Briefly, Tf was thiolated using 2-iminothiolane (Traut's reagent). Tf was dissolved in 0.1 M Na-borate buffer/0.1 mM EDTA, pH 8, followed by the addition of Traut's reagent (1 : 40 mol/mol). After incubation for 60 min at room temperature, thiolated Tf was immediately used for conjugation with SNALPs-Mal overnight at room temperature. The excess of Tf was removed by molecular exclusion chromatography, Sepharose CL-B4 column.

### 2.3. SNALPs Characterization: Mean Diameter, Polydispersity Index, and Zeta Potential

The mean diameter and size distribution of SNALPs were determined at 20°C by photon correlation spectroscopy (PCS) (N5, Beckman Coulter, Miami, USA). Each sample was diluted in deionized/filtered (0.22 *μ*m pore size, polycarbonate filters, MF-Millipore, Microglass Heim, Italy) water and analysed with detector at 90° angle. As measure of the particle size distribution, polydispersity index (PI) was used. For each batch, mean diameter and size distribution were the mean of three measures. For each formulation, the mean diameter and PI were calculated as the mean of three different batches. The zeta potential (ZP) of the SNALPs was determined in distilled water at 20°C by Zetasizer Nano Z (Malvern, UK). For each batch, mean diameter, size distribution, and ZP were the mean of three measures.

### 2.4. miR-34a Encapsulation into SNALPs Formulations

The amount of miRNA encapsulated into the SNALPs was measured spectrophotometrically. Briefly, 10 *μ*L of SNALPs suspension was dissolved in 990 *μ*L of methanol and analysed at 260 nm. Actual loading was calculated as amount (mg) of miRNA/mg of mg total lipids. The amount of miRNA loaded into the nanocarriers was expressed as miRNA actual loading and encapsulation efficiency, calculated as mg of miRNA/mg of total lipids and percent ratio between miR actually loaded into SNALPs and miR theoretical loading, respectively. For each batch, the results were the mean of three measures. For each formulation, the results were calculated as the mean of the measures obtained in three different batches (*n* = 3). The phospholipid content of the carrier suspension was determined by the Stewart assay [29]. Briefly, an aliquot of the SNALPs suspension was added to a two-phase system, consisting of an aqueous ammonium ferrothiocyanate solution (0.1 N) and chloroform. The concentration of DSPC was obtained by measuring the absorbance at 485 nm into the organic layer. The concentration of the total lipid content was calculated considering a constant ratio between the lipids.

### 2.5. Animals and *In Vivo* Models of Human MM

Male CB-17 severe combined immunodeficient (SCID) mice (6- to 8-week old; Harlan Laboratories, Inc., Indianapolis) were housed and monitored in our Animal Research Facility. All experimental procedures and protocols had been approved by the Institutional Ethical Committee (Magna Graecia University) and conducted according to protocols approved by the National Directorate of Veterinary Services (Italy). In accordance with institutional guidelines, mice were sacrificed when their tumors reached 2 cm in diameter or in the event of paralysis or major compromise in their quality of life, to prevent unnecessary suffering. For our study 25 SCID mice were inoculated in the interscapular area (sc) with 5 × 10^6^ MM cells in 100 *μ*L RPMI-1640 medium. After detection of palpable tumors, approximately 3 weeks following injection of MM cells, animals were randomized into 5 groups including 5 mice per group, and treatments were initiated. Each animal received a dose of 20 *μ*g of miR-34a SNALPs formulation. The treatment schedule included 5 treatments, three days apart, via tail vein. The tumor sizes were measured every two days until the day of first mouse sacrifice, using a caliper, and volume was calculated using the formula *V* = 0.5 × *a* × *b* ^2^, where *a* and *b* are the long and short diameters of the tumor, respectively.

### 2.6. Survival Analysis and Kaplan-Meier Plot

Survival was evaluated from the first day of treatment until death or sacrifice of animals. The observations were followed until last animal death or the tumors reached 2 cm in diameter according to our institutional guidelines. Percent of mice that survive with respect to the totality of animals included in each group is calculated and used to plot the survival curves (Kaplan-Meier curve).

### 2.7. FACS Analysis of TfR Expression in SKMM-1 Multiple Myeloma Cells

For determination of cell surface expression of TfR, fluorescence-activated cell sorting (FACS) analysis was performed using indirect staining of TfR (CD71). Briefly, we have seeded 50,000 SKMM-1 cells/well in 6 multiwell plates and incubated at 37°C for 24, 48, and 72 h. At the established times, the cells were collected, counted in a Burker chamber, and centrifuged at 1,300 rpm for 5 min at 4°C in order to remove the medium. The cells were suspended in PBS in order to obtain 500,000 cells for each time point. After another centrifugation at 2,000 rpm at 4°C for 5 min, the cells were suspended in 4% paraformaldehyde and incubated for 15 min at 4°C in the dark. Thereafter, after another centrifugation the cells were suspended in PBS/BSA (1% w/v) and incubated for 10 min at 4°C. Thereafter, the cells were incubated with a primary mouse monoclonal antibody raised against human TfR (CD71, H68.4, dilution: 1 : 100; Santa Cruz Biotechnology, Santa Cruz, CA, USA) or with an irrelevant immunoglobulin (IgG1) or in PBS overnight at 4°C. After incubation with primary antibody, the cells were again centrifuged and incubated with the secondary antibody (anti-mouse-FITC), in the dark for 1 h at 4°C. After washing, FACS sorting was performed using a FACScan (Becton Dickinson, Mountain View, CA, USA), and analysis was performed using CellQuest 2.0 (Becton Dickinson).

### 2.8. Statistical Analysis

Student's *t*-test, two-tailed test, and Log rank test were used to calculate all reported *P* values using GraphPad software (http://www.graphpad.com/). Graphs were obtained using SigmaPlot version 11.0.

## 3. Results and Discussion

**Figure 1:**
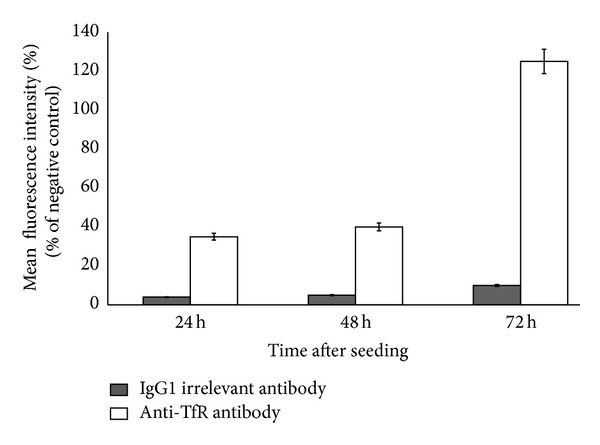
FACS analysis of the surface expression of TfR in SKMM-1 cells. SKMM-1 cells were cultured for 24, 48, and 72 h and the expression of CD71 (TfR) was evaluated at FACS as reported in Section 2. The intensity of TfR expression was represented as % of mean fluorescence intensity (MFI) calculated by comparing the MFI of cells incubated with antibodies with that of cells incubated in PBS. A significant increase of MFI was observed in cells labelled with anti-TfR (empty squares) if compared with that of cells labelled with irrelevant IgG1 (full squares) suggesting a significant expression of TfR on the surface of SKMM-1 cells. Each value was the mean of at least three different determinations performed in three different experiments. Bars, SEs.

In this study, we investigated the possible use of miR-34a in the treatment of MM. We have previously investigated the activity of miR-34a in different preclinical models using a commercial available lipidic emulsion [28]. Moreover, we previously developed SNALPs encapsulating miR-34a to treat medulloblastoma cells *in vitro* [21]. Here we proposed the use of SNALPs encapsulating miR-34a to treat MM *in vivo*. We also investigated the possibility to modify the previously developed SNALPs in order to obtain enhanced antitumor activity. To do this, as first strategy, we encapsulated a chemically modified miRNA into the SNALPs. In particular, OMet miR-34a was selected due to substantial degree of nuclease resistance and high binding affinity with the target [30]. A second strategy consisted of Tf conjugation with SNALPs in order to enhance targeting and delivery to cancer cells *in vivo*. In fact, SKMM-1 MM cells (used in our study) expressed on their surface significantly high levels of the TfR as demonstrated by the analysis of TfR expression performed during FACS analysis (Figure 1). Interestingly, the expression of the receptor increased in a time-dependent manner during the culture of SKMM-1 cells suggesting its role in the survival of these cells. Moreover, MM cells are characterized by an over-expression of the Tf receptor [33, 34]. On the basis of these data and on the known high rate of internalization of the TfR, we were prompted to develop SNALPs armed with Tf for the treatment of MM.

In Table 1, the developed formulations, as well as their characteristics in terms of mean diameter, PI, and ZP, are reported. SNALPs have a mean diameter ranging between about 128 and 158 nm; the use of an OMet miRNA slightly decreased mean diameter. All the formulations have a narrow size distribution, although a slight increase from 0.16 to 0.25 was observed when encapsulating an OMet miR-34a.

In Table 2, the amount of miR-34a encapsulated into the SNALPs is reported. All the formulations displayed a high amount of miR-34a encapsulated into the SNALPs. In particular, wild type miR-34a was encapsulated with an efficiency of about 80%. This result was not surprising and in agreement with other previously developed SNALPs formulations [17, 21]. Interestingly, the use of an OMet miRNA led to a significant increase of the encapsulation efficiency (about 98% of the miRNA initially used in the formulation). The interaction between the negative charge of nucleic acid and the positive one of cationic lipid is the driving force for the encapsulation of nucleic acids into the SNALPs [17]. However, an OMet miR-34a, if compared to a wild type miR-34a, is more lipophilic, due to the presence of the methyl residue bound to 2′-position of the sugar moiety of each nucleotide. Therefore, in our study, an additive hydrophobic interaction between the miRNA and the lipid bilayer can represent an additional contribution to high miRNA loading into the SNALPs, when encapsulating a completely 2′-O methylated miRNA.

Thereafter, we modified the surface of SNALPs with Tf in order to achieve an additional improvement of the delivery in MM cells. SNALPs modified with targeting ligands have previously been reported for glioblastoma cells [31]. In the study from Costa et al., SNALPs were modified by postinsertion method. In the present work, we preferred to use a different approach and Tf was coupled to preformed SNALPs. Tf binding to the nanoparticle surface did not significantly affect the size characteristics of the vesicles. On the contrary, a slight decrease of the PI, from 0.16 to 0.14 and from 0.25 to 0.21, was observed for both SNALP1 and SNALP2, respectively. This slight increase of PI can be explained with the further purification step after the conjugation with Tf. Tf-SNALPs also showed a significant decrease of the zeta potential (Table 1), as expected by the presence of the protein on the vesicle surface. The Tf conjugation on the preformed SNALPs did not affect miRNA encapsulation into the vesicles (data not shown).

**Figure 2:**
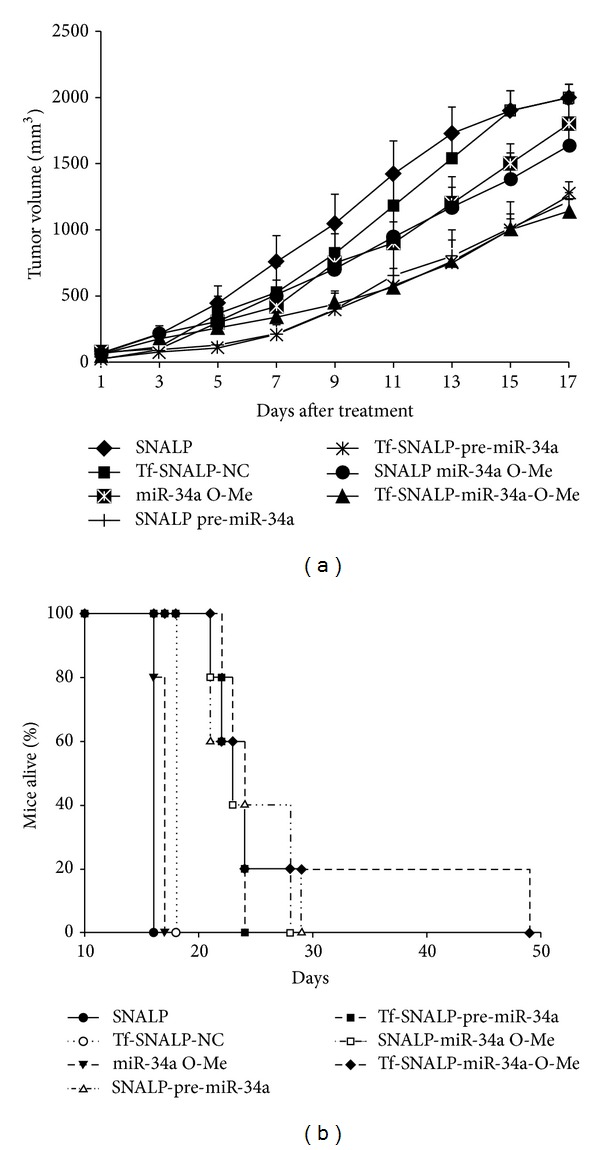
Systemic delivery of miR-34a SNALPs formulations inhibits growth of human MM tumors in mice. (a) Mice carrying palpable subcutaneous SKMM-1 tumor xenografts were treated with 20 *μ*g of pre-miR-34a or miR-34a OMet SNALPs or Tf-SNALPs formulations by intravenous tail vein injections. Caliper measurement of tumors was taken every 2 days from the day of first treatment. Averaged tumor volumes of 5 mice per group are reported ± SD. (b) Survival curves (Kaplan-Meier) of treated mice show prolongation of survival after formulated miR-34a treatment compared to controls (log-rank test, *P* < 0.005). Survival was evaluated from the first day of treatment until death or sacrifice.

We next explored the effects of the systemic delivery of miR-34a SNALPs formulations in controlling the growth of SKMM-1 xenografts. When subcutaneous MM tumors became palpable, mice were randomized and systemically treated, via tail vein, with different miR-34a or miR-NC SNALPs formulations at a concentration of 1 mg miR-34a/kg per mouse. Following 5 injections (3 days apart), a significant antitumor effect of miR-34a SNALPs formulation (SNALP 1) versus the control was detected (Figure 2(a)). The treatment with plain SNALPs did not elicit any effect on the tumor growth (Figure 2(a)). The use of SNALPs encapsulating OMet miR-34a resulted in a lower inhibition of tumor growth, compared to SNALPs encapsulating wild type miR-34a. If we consider that the ONs modification with a 2′-O-methyl group maintains a strong base-pairing with target [32], a different delivery mechanism of the miRNA from the vesicle could be responsible for the lower antitumor activity when using SNALPs encapsulating OMet miR-34a. The modification of Tf conjugation with SNALPs affected miRNA antitumor activity, depending on the RNA chemistry. In particular, the SNALPs conjugation with Tf significantly increased the effect of OMet miR-34a on tumor growth (Figure 2(a)). In particular, after 9 days from the beginning of the treatment, the effect of Tf-SNALPs encapsulating OMet miR-34 was comparable to that obtained in animal treated with SNALPs encapsulating wild type miR-34a. Surprisingly, conjugation with Tf had no effect on SNALPs encapsulating wild type miR-34a. The increased efficacy of the treatment, observed in the case of Tf-SNALP2, can be explained with a higher interaction and uptake of SNALPs in MM cells, characterized by an overexpression of TfR (see also Figure 1) [33, 34]. The importance of the TfR for the development of malignant human hematopoietic has been well established [35]. Moreover, in the case of metastasis originated by the MM cells, an increased growth of vessel characterizes the tumor mass. It has been demonstrated that SNALPs predominantly deliver siRNA to areas adjacent to functional tumor blood vessels [36]. Therefore, in our study, a targeting of tumor vessel, characterized by an enhanced expression of the TfR, can also be hypothesized. It is worthy of note that the use of Tf was beneficial only in the case of SNALPs encapsulating wild type miR-34a. To justify these findings, we hypothesize that the animal treatment with SNALP1 led to the highest miRNA delivery, in these experimental conditions. For this reason, animal treatment with Tf-SNALP1 did not result in an increased inhibition of the tumor growth; in line with this hypothesis, the use of Tf-SNALP2 led to an increased inhibition of the tumor growth, which was similar to that obtained in animals treated with SNALP1 and Tf-SNALP1.

In Figure 2(b), the mice survival following treatment with the different SNALP-based formulations is reported. All the animals treated with plain SNALPs died after 17 days of treatment. Mice survival was prolonged with all the SNALP-based formulations encapsulating miR-34a. In particular, mice treatment with SNALPs containing wild type miR-34a or OMet miR-34a resulted in mice survival until 28-29 days from the beginning of the treatment. The conjugation of Tf with SNALPs containing wild type miR-34a did not prolong mice survival. Surprisingly, the use of Tf-conjugated SNALPs encapsulating OMet miR-34a resulted in a significant prolongation of the mice survival until 49 days. This could be explained with the higher biological stability of OMet miR-34a that can result in the highest persistence of the miR-34a into the cells. Of course, the superiority of the formulation Tf-SNALP2 was not observed in the experiments of tumor growth, where the animals were sacrificed, according with the institutional guidelines, following 17 days from the beginning of the treatment.

## 4. Conclusions

In this work, we developed SNALPs to deliver miR-34a. SNALPs were also modified to enhance miR-34a delivery in cancer. The two strategies used, the encapsulation of a chemically modified OMet miR-34a and the binding Tf on the nanoparticle surface, did not affect the technological properties of the nanocarriers. The antitumor effect of miR-34a was demonstrated in an experimental animal model of MM. A significant inhibition of the tumor growth was observed in animals treated with all the formulations. However, only the combination of OMet miR-34a and Tf-conjugation with SNALPs resulted in a significant prolongation of the mice survival.

This study demonstrated that miR-34a represents a powerful tool to treat tumors as MM, for which present treatment can only prolong the survival. However, this kind of therapy will need to develop *ad hoc* delivery systems in order to optimize the anticancer activity of miRNAs. As shown in this work, chemical modifications aimed to improve the biological stability of the miRNA that could be successfully combined with targeting approaches based upon the active delivery of the nanotechnological devices. This approach can be reasonably extended to deliver miRNA and other nucleic acids in other tumors characterized by over-expression of TfR. Additional studies are required to investigate if the developed treatment can be associated to toxicity in healthy tissue.

## Figures and Tables

**Table 1:** Mean diameter, polydispersity index, and zeta potential of the different SNALPs-based formulations.

Formulations	miRNA chemistry	Targeting ligand	Mean diameter (nm) ± SD	PI ± SD	ZP (mV) ± SD
SNALP1	Wild type	—	157.18 ± 17.18	0.16 ± 0.03	−13.52 ± 2.28
SNALP2	OMet	—	127.75 ± 10.68	0.25 ± 0.03	−5.19 ± 5.38
Tf-SNALP1	Wild type	Tf	158.32 ± 10.25	0.14 ± 0.05	−23.62 ± 1.54
Tf-SNALP2	OMet	Tf	130.84 ± 8.41	0.21 ± 0.02	−18.13 ± 4.39

**Table 2:** miRNA encapsulation into the SNALPs.

Formulations	Actual loading (*μ*g miRNA/mg lipids)	Encapsulation efficiency (%)
SNALP1	160.0 ± 4.8	80 ± 3
SNALP2	196.5 ± 10.6	98 ± 5

Theoretical loading: 200 *μ*g miRNA/mg lipids.
